# Set-up of a pharmaceutical cell bank of *Magnetospirillum gryphiswaldense* MSR1 magnetotactic bacteria producing highly pure magnetosomes

**DOI:** 10.1186/s12934-024-02313-4

**Published:** 2024-02-28

**Authors:** Théo Chades, Raphaël Le Fèvre, Imène Chebbi, Karine Blondeau, François Guyot, Edouard Alphandéry

**Affiliations:** 1 Nanobacterie SARL, 36 Boulevard Flandrin, 75116 Paris, France; 2 Institut de biologie intégrative de la cellule, UMR 9198, Université Paris Saclay, 1 Av. de la Terrasse, 91198 Gif sur Yvette, France; 3 Institut de minéralogie de physique des matériaux et de cosmochimie UMR 7590, Sorbonne Université, Université Pierre et Marie Curie, Muséum National d’Histoire Naturelle, 4 Place Jussieu, 75005 Paris, France

**Keywords:** *Magnetospirillum gryphiswaldense* MSR1, Magnetosome, Cell Bank, Minimal medium, Fed-batch cultivation, Scale-up, Magnetotactic bacterium, Pharmaceutical grade nanoparticle

## Abstract

We report the successful fabrication of a pharmaceutical cellular bank (PCB) containing magnetotactic bacteria (MTB), which belong to the *Magnetospirillum gryphiswaldense* MSR1 species. To produce such PCB, we amplified MTB in a minimal growth medium essentially devoid of other heavy metals than iron and of CMR (Carcinogenic, mutagenic and reprotoxic) products. The PCB enabled to acclimate MTB to such minimal growth conditions and then to produce highly pure magnetosomes composed of more than 99.9% of iron. The qualification of the bank as a PCB relies first on a preserved identity of the MTB compared with the original strain, second on genetic bacterial stability observed over 100 generations or under cryo-preservation for 16 months, third on a high level of purity highlighted by an absence of contaminating microorganisms in the PCB. Furthermore, the PCB was prepared under high-cell load conditions (9.10^8^ cells/mL), allowing large-scale bacterial amplification and magnetosome production. In the future, the PCB could therefore be considered for commercial as well as research orientated applications in nanomedicine. We describe for the first-time conditions for setting-up an effective pharmaceutical cellular bank preserving over time the ability of certain specific cells, i.e. *Magnetospirillum gryphiswaldense* MSR1 MTB, to produce nano-minerals, i.e. magnetosomes, within a pharmaceutical setting.

**Supplementary Information:**

The online version contains supplementary material available at 10.1186/s12934-024-02313-4.

## Introduction

Magnetotactic bacteria (MTB) belong to a polyphyletic group of bacteria with the distinctive feature of producing intracellular magnetic nanoparticles called magnetosomes [10, 14, 33]. MTB are ubiquitous in aquatic environments and are preferentially found in sediments and water columns in the oxic-anoxic transition zone and the anoxic region [22, 37, 58]. These habitats have a specific chemical stratification that favors magnetosome biosynthesis in terms of iron availability and oxygen levels, since biomineralization occurs in microaerobic or anaerobic conditions [21, 37]. Magnetosomes are subcellular organelles consisting of an iron oxide or sulfide crystal enveloped in a phospholipid bilayer membrane associated with specific proteins [1, 24]. They are almost always organized in a unique chain that allows passive alignment of the bacteria along the Earth magnetic field lines [32].

Magnetite magnetosomes as biogenic iron oxide nanoparticles have several benefits over their chemical counterparts, which have been described as having some potential for medical applications [6, 7], but require complex, toxic and/or non-fully reproducible synthesis conditions such as those involving organic solvents, high temperatures/pressures and additional steps to stabilize and vectorize the particles [35]. These advantages derive from their highly regulated synthesis thanks to a specific set of genes, resulting in a high chemical purity, narrow size distribution and stable ferrimagnetic monodomain. These 30 magnetosome-specific genes are organized in 4 operons clustered in a magnetosome island (MAI) [55, 71]. Their unique properties in addition to their biocompatibility [11, 15, 16] make magnetosomes attractive nanostructure candidates for biomedical and biotechnological applications such as contrast agents in magnetic resonance imaging (MRI) imaging [39], diagnostic and detection tools or targeted drug delivery systems with functionalized membranes [4, 5, 8, 9, 34, 72]. Another promising application is cancer treatment using local moderate hyperthermia induced after intra-tumoral injection of magnetosomes and application of an external energy source such as laser [53] or alternative magnetic field [36].

However, the commercial use of magnetosomes first requires scaling up magnetosome production. Despite facing certain hurdles [12], this objective was achieved with several MTB such as *Magnetospirillum gryphiswaldense* MSR1, *Magnetospirillum magneticum* AMB1, *Magnetospirillum* ME1 and *Magnetovibrio blakemorei* MV1, which were shown to produce more than 10 mg of magnetosomes per liter of medium under specific optimized conditions [2]. Large-scale batch culture of magnetotactic bacteria was also reported for the strain MG-T1 in a 1000 L growth medium, yielding the production of 2.6 g of magnetosomes after 4 days [43]. The highest yield of magnetosome production was achieved with *Magnetospirillum gryphiswaldense* strain MSR1 using a pH–stat fed-batch strategy, resulting in 356.52 mg of dried magnetosomes per liter of growth medium [76]. The second challenge lies in being able to produce pure magnetosomes. This is not a natural outcome of MTB production. Indeed, under amplification in a non-depleted and non-pharmaceutical grade medium, these microorganisms have been shown to yield the incorporation in magnetosomes of certain other heavy metals than iron such as manganese, zinc, cobalt and copper [49, 68]. Such medium should probably be avoided for pharmaceutical applications. On the one hand, it leads to a reduction in magnetosome iron content relatively to other heavy metals and to some variations in magnetosome crystalline structure, mean size, coercivity, anisotropy, heating properties [3, 41, 42], depending on the exact content of such medium. On the other hand, it contains carcinogenic, mutagenic and reprotoxic chemicals, heavy metals other than iron and products of unknown composition [56, 76], which are not recommended by pharmaceutical standards above certain threshold values as specified in ICH-Q3D guidelines [18]. In our case, we have therefore developed a minimal pharmaceutically acceptable growth medium to amplify *Magnetospirillum gryphiswaldense* MSR1 and produce highly pure magnetosomes [13, 50], hence abiding by such international recommendations.

As an extension of our previous work [13, 50], we report here the development of a stable pharmaceutical cell bank (PCB) of MSR1 magnetotactic bacteria, which can be used to produce highly pure magnetosomes *‘‘on demand’’*. Such cell bank appears essential to reduce the costs and delays induced by launching MTB growth from a commercial cell bank (CCB), to provide a genetically stable and pure starting inoculum for reproducible production processes [27, 64], and to yield purer magnetosomes with the PCB than with the CCB. Here, we follow the rules of the cell banking system, which stipulates that a cell bank should first consist of cell banks of one or two levels [19, 61], second comply with pharmaceutical standards, e.g. in terms of pharmaceutical types of medium used for bacterial amplification [18, 20], third be identical to the original MTB strain, pure, and stable according to ICH-Q5D criteria [19, 63], fourth be preservable over time while maintaining its stability [31, 65]. To add even more value to our PCB, we demonstrate that it can be prepared under conditions of high cell density and cryopreserved as such. This allows to launch a large scale MTB cultivation, simply by starting with an inoculum of the PCB, hence fulfilling the desired main function of the PCB [48].

## Materials and methods

### Strain

*Magnetospirillum gryphiswaldense* strain MSR1 (DSM6361) was purchased from Deutsche Sammlung von Mikroorganismen und Zellkulturen (DSMZ Brunswick, Germany) and stored in aliquots of 1 mL at − 80 °C as cryo-stocks and labeled as Control Cell Bank (CCB).

### Media composition for cell bank culture and highly pure magnetosome production

#### Composition of the non-minimal growth medium used to prepare the commercial cell bank (CCB)

Per liter, the growth medium of the commercial cell bank used by DSMZ to cultivate *Magnetospirillum gryphiswaldense* MSR1 magnetotactic bacteria (“DSMZ—Medium 380,” n.d.) consisted of 0.68 g of KH_2_PO_4_, 0.12 g of NaNO_3_, 0.37 g of L( +)-tartaric acid, 0.37 g of succinic acid, 0.05 g of Na-acetate, 0.1 g of yeast extract, 5 mL of modified Wolin mineral solution, 2 mL of Fe(III) quinate solution (0.01 M), 0.5 mL of sodium resazurin (0.1% w/v), 0.05 g of Na-thioglycolate and 1 mL of a vitamin solution containing 7 different vitamins (composition presented below). The pH of such medium was adjusted to 6.75 by addition of NaOH 1 M. Per liter, the modified Wolin mineral solution consisted of 1.5 g of nitrilotriacetic acid, 3 g of MgSO_4_,7H_2_O, 0.5 g of MnSO_4_,H_2_O, 1 g of NaCl, 0.1 g of FeSO_4_.7H_2_O, 0.18 g of CoSO_4_.7H_2_O, 0.1 g of CaCl_2_.2H_2_O, 0.18 g of ZnSO_4_.7H_2_O, 0.01 g of CuSO_4_.5H_2_O, 0.02 g of AlK(SO_4_)_2_.12H_2_O, 0.01 g of H_3_BO_3_, 0.01 g of Na_2_MoO_4_.2H_2_O, 0.03 g of NiCl_2_.6H_2_O, 0.3 mg of Na_2_SeO_3_.5H_2_O and 0.4 mg of Na_2_WO_4_.2H_2_O. The pH of the modified Wolin mineral solution was firstly adjusted to 6.5 after nitrilotriacetic acid dissolution by adding first KOH 1 M. Secondly, pH was adjusted to pH 7.0 after dissolution of all remaining components by adding KOH 1 M. Per liter, the 7 vitamins solution consisted of 0.1 g of vitamin B12, 0.08 g of *p*-aminobenzoic acid, 0.02 g of D-( +)-biotin, 0.2 g of nicotinic acid, 0.1 g of Ca-pantothenate, 0.3 g of pyridoxine hydrochloride and 0.2 g of thiamine-HCl.2H_2_O. Per liter, the Fe(III) quinate solution 0.01 M consisted of 4.5 g of FeCl_3_.6H_2_O and 1.9 g of quinic acid.

#### Composition of the minimal growth medium used to prepare the pharmaceutical cell bank (PCB)

Per liter, the pharmaceutical cell bank growth medium used to grow *Magnetospirillum gryphiswaldense* MSR1 magnetotactic bacteria consisted of 2.6 g of Na-lactate, 0.4 g of NH_4_Cl, 0.1 g of MgSO_4_,7H_2_O, 0.5 g of K_2_HPO_4_, 5 mL of ferric citrate solution, 0.1 mL of a vitamin solution containing 9 different vitamins and 0.5 mL of a mineral elixir solution. Per liter, the ferric citrate solution consisted of 5.259 g of ferric citrate. Per liter, the vitamin solution containing 9 different vitamins consisted of 0.002 g of biotin, 0.4 g of Ca-pantothenate, 0.002 g of folic acid, 2 g of inositol, 0.4 g of nicotinic acid, 0.2 g of *p*-aminobenzoic acid, 0.4 g of pyridoxine HCl, 0.2 g of riboflavin and 0.4 g of thiamine HCl. Per liter, the mineral elixir solution consisted of 39.74 g of CaCl_2_,2H_2_O and 1 g of FeSO_4_,7H_2_O. All chemicals were purchased in pharmaceutical grade from Merck (Darmstadt, Germany).

#### Composition of the minimal growth medium used to store the PCB.

The medium used to store the PCB consisted of the minimal growth medium describe in the above section supplemented with 5% (w/v) of dimethylsulfoxyde (DMSO).

#### Composition of the minimal pre-growth medium used for the pre-growth steps in 7.5 L bioreactor culture

The pre-growth minimal medium composition is the same as the PCB minimal medium composition except that it does not contain iron citrate.

#### Composition of the minimal growth medium used for the growth step in 7.5 L bioreactor culture

Per liter, the growth minimal medium consisted of 1.3 g of Na-lactate, 0.223 g of NH_4_Cl, 0.027 g of MgSO_4_,7H_2_O, 0.067 g of K_2_HPO_4_, 0.067 mL of 9 vitamins solution and 0.08 mL of mineral elixir solution. Per liter, the vitamin solution containing 9 different vitamins consisted of 0.002 g of biotin, 0.4 g of Ca-pantothenate, 0.002 g of folic acid, 2 g of inositol, 0.4 g of nicotinic acid, 0.2 g of *p*-aminobenzoic acid, 0.4 g of pyridoxine HCl, 0.2 g of riboflavin and 0.4 g of thiamine HCl. Per liter, the mineral elixir solution consisted of 39.74 g of CaCl_2_,2H_2_O and 1 g of FeSO_4_,7H_2_O. All chemicals were purchased in pharmaceutical grade from Merck (Darmstadt, Germany).

#### Composition of the minimal feed medium used for the growth step in 7.5 L bioreactor culture

Per liter, the feed minimal medium consisted of 100 g of lactic acid, 4.77 g of NH_3_, 2.4 g of MgSO_4_,7H_2_O, 6 g of K_2_HPO_4_, 2 g of FeCl_3_,6H_2_O, 1 mL of 9 vitamins solution and 7 mL of mineral elixir solution. Per liter, the vitamin solution containing 9 different vitamins consisted of 0.002 g of biotin, 0.4 g of Ca-pantothenate, 0.002 g of folic acid, 2 g of inositol, 0.4 g of nicotinic acid, 0.2 g of *p*-aminobenzoic acid, 0.4 g of pyridoxine HCl, 0.2 g of riboflavin and 0.4 g of thiamine HCl. Per liter, the mineral elixir solution consisted of 39.74 g of CaCl_2_,2H_2_O and 1 g of FeSO_4_,7H_2_O. All chemicals were purchased in pharmaceutical grade from Merck (Darmstadt, Germany).

### Conditions of cultivation of magnetotactic bacteria and magnetosome preparation

#### First culture condition: High cell load pharmaceutical cell bank production in 3L bioreactor batch culture

The PCB was produced in batch culture in a 3L bioreactor filled with 0.5 L of PCB minimal growth medium. Cultures were performed in triplicate. Before inoculation with 5 mL of CCB cryo-stock, the medium was sparged with a gas mixture of O_2_/N_2_ (2/98%) for 20 min and the temperature was pre-adjusted at 29.5 °C. Batch cultivation was conducted for 6 days at 29.5 °C with a fixed agitation of 110 rpm. The pO_2_ concentration and pH were monitored throughout cultivation to maintain microaerobic conditions (Additional file 1: Figure S1). The pO_2_ concentration was maintained below 2% vol. by bubbling O_2_/N_2_ (2/98%) gas if necessary. During the first phase of culture, the pH was stable at around 7 and the oxygen partial pressure was maintained at around 20 mbar (∼ 2% vol.) to promote cell growth without compromising magnetosome synthesis by sparging the culture every 24 h [28] and prevent oxygen accumulation in the large headspace volume (1/5 v/v) that could occur while stirring the growth medium due to gas–liquid interface transfer under low bacterial oxygen uptake [60]. During the second phase of cultivation, the oxygen partial pressure decreased to 0.25% sat. from 118 h to the end of culture (Additional file 1: Figure S1), indicating that the biomass had reached a point where the oxygen uptake rate became higher than the oxygen transfer rate. Concomitantly, the pH increased rapidly, indicating a rapid biomass growth rate. After 142 h of cultivation, a slowing of the pH increase indicated the end of the exponential growth phase. Previous test cultures for cell bank production with biomass sampling confirmed the relationship between lactate uptake, pH evolution and growth.

The final OD_565_ reached 0.279 with a generation time of 19.93 h (Additional file 1: Figure S1). Using the Petroff hemocytometer, a correspondence factor of 9.10^8^ cells/mL for an OD_565_ of 1 gives a final bacterial concentration of 2.5.10^8^ cells/mL.

After cultivation, MSR1 cells were centrifuged at 4000 rpm for 45 min (GR4i Jouan Centrifuge, Thermo). Pellet was resuspended in fresh PCB storage minimal medium supplemented with DMSO to reach a concentration of 9.10^8^ cells/mL, i.e. ∼ OD_565_ of 1 according to a high cell density cryopreservation strategy. Cryotubes were labeled as PCB and frozen at − 80 °C prior their used for PCB characterization experiments and as starting inoculum for production of highly pure magnetosomes during 7.5 L fed-batch cultures experiments.

#### Second culture condition: Subculturing experiment in 150 mL bottle

1 mL of PCB were subcultured 24 times in 50 mL of PCB minimal medium in 150 mL bottles at 29.5 °C and 110 rpm (Thermo Scientific, MaxQ 2000) for 3 to 5 days. Medium was not flushed and showed classic atmospheric gas composition and initial aerobic conditions before bacterial uptake to microaerobic. After each subculture, the cell bank medium was discarded and replaced by fresh medium to reach a concentration of 9.10^7^ cells/mL (~ OD565 of 0.1). This experiment was performed in sextuplet and used to characterize PCB stability over several bacterial generations.

#### Third culture condition: MTB cultivation on LB agar plates

A volume of 1 mL of CCB and PCB bacterial suspensions were deposited on top of LB agar plates, which were then incubated at 29.5 and 37 °C for 3 days. LB agar was prepared using 20 g of LB broth and 15 g of agar per liter and served to detect bacterial contaminants that if present should develop as observable colonies of microorganisms. This experiment was used to characterize the purity of the PCB.

#### Fourth culture condition: Fed-batch culture in 7.5 L bioreactor using minimal medium to produce highly pure magnetosomes starting from the PCB

During the first pre-growth step (preculture 1, PC1), 450 µL of unfrozen CCB, of unfrozen PCB or of 16 months stored PCB were grown in 250 mL of pre-growth minimal medium in 500 mL square bottles and incubated for 8 days at 29.5 °C. On the last day, PC1 were agitated in an orbital shaker (Thermo Scientific, MaxQ 2000) operating at 110 rpm. During the second pre-growth step (preculture 2, PC2), 500 mL of PC1 were transferred in a 3L bioreactor (Applikon) filled with 1.5 L of pre-growth minimal medium and incubated for 2 days at 29.5 °C under agitation at 110 rpm. These pre-growth steps were carried out in the absence of iron and in the presence of an initial quantity of oxygen until depletion to microaerobic conditions. During the pH–stat fed-batch culture inspired from [76], a volume of PC2 was transferred in a 7.5 L automatized bioreactor (Applikon) containing 4L of minimal growth medium to achieve an initial OD_565_ of 0.11 ± 0.01. The pO_2_ was kept below 0.1% vol. by controlling airflow and stirring rates between 20 to 107 mL/min and 150 to 250 rpm respectively after initial oxygen depletion during the first 12 h of culture. The temperature was maintained at 29.5 °C and pH was kept at 6.84 by addition of an acidic feed medium (pH = 2.99 ± 0.02). All these parameters were adjusted and monitored using an *ez*-Control controller (Applikon) and BioXpert software (Applikon). This growth step was carried out under microaerobic conditions in presence of iron. After cultivation ended at 140 h, the bacterial culture was washed and concentrated using a tangential filter column (MiniKros Sampler, PES 0.2 µm) to a volume of 1 L and stored at − 80 °C before magnetosome extraction. Cultures were performed in duplicate. Measurement of cell density (OD_565_), dry cell weight and magnetic response were performed on bacterial samples harvested at 0, 72, 96, 120 and 140 h of cultivation.

This experiment was used to characterize PCB stability over long term storage at − 80 °C and to compare PCB and CCB performances. In addition, the purity of magnetosomes produced with the PCB was investigated.

#### Magnetosomes extraction

Bacterial concentrates of 7.5 L PCB fed-batch cultures were thawed and diluted with deionized water to reach a final OD_565_ of 20. Cells were lysed in KOH 2 M agitated at 150 rpm with a stirring blade for 1 h at 80 °C. Lysate was then placed against a Neodymium magnet overnight to separate magnetosomes from the supernatant containing cell debris. The liquid phase was gently removed using a vacuum pump. Magnetosomes were washed 2 times with 10X phosphate-buffered saline (PBS) and then 3 times with deionized water by magnetic selection against a Neodymium magnet. Subsequently, magnetosomes were collected in 50 ml conical tubes and centrifuged at 4000 g for 45 min at 6 °C (Eppendorf, Centrifuge 5810 R) and the supernatant was disposed of. Conical tubes were kept at − 80 °C for 48 h. Extracted frozen magnetosomes were lyophilized at − 50 °C and 0.003 mbar for 48 h using a freeze drier (Labconco, Free Zone^®^ 70020 2.5 L). Freeze-dried magnetosomes were ground to obtain a raw powder and finally weighed to determine the yield in dry weight of magnetosomes for each culture.

### Cell bank characterization

#### ***Measurement of cell density (OD***_***565***_***), dry cell weight, cell length, cell numeration and magnetic response***

Optical density at 565 nm (OD_565_) of a suspension containing MTB mixed in 1 mL was measured using an UV–visible spectrophotometer (Secomam, UviLine 9400). To determine dry cell weight (DCW), 20 mL of bacterial sample from MSR-1 culture were centrifuged at 4000 rpm for 35 min at 4 °C (Eppendorf, Centrifuge 5810 R). Pellets were kept at − 80 °C for 48 h and lyophilized at − 50 °C and 0.003 mbar for 48 h using a freeze drier (Labconco, Free Zone^®^ 70020 2.5 L). Dry pellets were weighed to determine DCW. Bacterial samples, after dilution to an OD_565_ of 0.5, were observed under an optical microscope (Zeiss, Primo Vert) to determine bacterial morphology, cell length considering 100 bacteria, cell concentration using a Petroff hemocytometer and magnetic response as described by Berny et al. [13].

#### Flow cytometry

After thawing, CCB and PCB samples were centrifugated at 12,000 g for 10 min (Eppendorf, miniSpin plus). A first sample of CCB and PCB was resuspended in phosphate-buffered saline (PBS) while a second was resuspended in ethanol 70% (v/v) and heated at 50 °C for 1 h. Pellets were subsequently recovered and resuspended in phosphate-buffered saline (PBS). Bacteria were stained with propidium iodide (PI) at a concentration of 100 ng/L and analyzed using a CytoFLEX (Beckman Coulter). Diluted samples were excited using a 488 nm laser and fluorescence for forward scatter (FSC) and side scatter (SSC) signals was measured, which reflect the distribution in bacterial size and granulometry, respectively. Cell viability was assessed using PI after excitation at 488 nm and fluorescence was detected using a 610/20 BP filter (FL8).

#### Transmission electron microscopy

Bacterial sample was diluted to an OD_565_ of 0.5 and centrifugated at 12,000 g for 10 min (Eppendorf, miniSpin plus). The cell pellet was washed 3 times with deionized water and kept at − 80 °C. After thawing, 5 µL of the bacterial suspension was deposited on a carbon-coated copper grid (300 mesh grid, Oxford Instruments) and dried for 3 h at room temperature. Grids were subsequently observed using a transmission electron microscope JEOL JEM-2100 operated at 200 kV. Magnetosome number per bacterium and magnetosome core size were measured manually on TEM micrographs obtained at × 4000 and × 12,000 magnification respectively, for 100 bacteria and 250 magnetosomes respectively, using ImageJ software.

#### PCR methods

For MSR1 strain identification, 5 mL of PCB were centrifuged at 4000 rpm for 30 min at 4 °C (Eppendorf, Centrifuge 5810 R) and suspended in 1 mL of deionized water. The suspension was heated at 95 °C for 20 min to obtain the DNA extract. PCR mix consists of 50 µL of PCR buffer, dNTP 200 µM, primers 0.2 µM, MgCl_2_ 1.5 mM and 0.2 µL of TaQ pol (Platinium Taq DNA Polymerase, Invitrogen). Selected P209 and P326 primer couples adapted from Guo et al. [25] were designed based on the MSR1 16S ribosomal sequence (accession number: CP027526.1). P209 primers couple consist of a 23 dNTP forward primer with a Tm of 68 °C (5′ GTACCGTCATCATCATCGTCCCC 3′) and a 20 dNTP reverse primer with a Tm of 66.4 °C (5′GTGAGGTAACGGCTCACCAA 3′). P326 primers couple consist of a 20 dNTP forward primer with a Tm of 66.5 °C (5′ GCGATTCCGACTTCATGCAC 3′) and a 20 dNTP reverse primer with a Tm of 65 °C (5′ TGGTGACTTGTCTTCGGACG 3′). Amplification was performed using a thermocycler GeneAmp^®^ PCR System 9700 (Applied Biosystems) by mixing 8.7 µL of PCR mix, 5 µL of PCB DNA extract and 40.3 µL of sterile deionized water with the following heating sequences: 1 min-94 °C, 1 min-68.4 °C and 2 min-72 °C for 35 cycles. Gel migration was performed at 120 V for 55 min on agarose gel 2% composed of 100 mL TAE buffer 1X, 2 g agarose and 10 µL SYBR Safe (Invitrogen). Gel revelation was performed using a BIO-RAD Gel Doc™ EZ imager. Negative and positive controls consist of sterile deionized water and CCB DNA (DSMZ 6361) respectively.

#### Genomic variant analysis

A variant analysis was performed between genomes from CCB and PCB. CCB DNA was purchased from DSMZ while PCB DNA was extracted using a MasterPure Gram Positive DNA Purification Kit (Biosearch Technologies, LGC). DNA sequencing and variant analysis were performed by the Next Generation Sequencing (NGS) Core Facility. Samples were prepared in accordance with the TruSeq genomic protocol using the sequencing kit NextSeq 500/550 High Output Kit v2 (Illumina). Sequencing was performed using a NextSeq NB552053 system (Illumina) with 50–34 sequencing cycles (paired-end). Collected data were sorted using bcl2fastq2-2.18.12 and adapter trimming was performed with Cutadapt 3.2. Quality control was carried out using FastQC v0.11.5. Mapping was performed using BWA 0.7.17-r1188 software with *Magnetospirillum gryphiswaldense* MSR1 genome as reference (accession number: CP027526.1). Genomic variations were detected using Freebayes v1.1.0 against the reference genome presented above. For each PCB sample, genomic variations already present in CCB genome sequencing were removed from the variations found. To assess the presence of a genetic variations in most bacteria, a filter on the allelic frequency of variations with a minimum coverage of 10 reads was applied with a level of 100 and 80%. This experiment was performed on three different preparations of PCB (PCB1, PCB2, PCB3).

#### Total intracellular iron assay

Total intracellular iron concentration was determined using an iron destructive colorimetric assay. Bacterial samples at 0, 72, 96, 120 and 140 h of cultivation were first concentrated or diluted to an OD_565_ of 2 in a final volume of 2 mL with deionized water. Pellets were recovered after centrifugation at 12,000 g for 10 min (Eppendorf, miniSpin plus), washed 3 times with deionized water and kept at − 80 °C. After thawing, cells were resuspended in 375 µL of HCl 12 N and placed at 50 °C overnight in a heating block (Type OBT2, Grant Instruments). A volume of 125 µL of HNO_3_ 11 N was added to the cell lysate. After 24 h at room temperature, samples were mixed with 500 µL of deionized water. 50 µL of the sample was mixed with 50 µL of H2O2 20% (v/v), vortexed. After 15 min, 850 µL of deionized water were added and then 50 µL of KSCN 2 M. Absorbance was measured at 476 nm using a spectrophotometer (Secomam, UviLine 9400). Iron concentrations were determined in accordance with a standard calibration curve (0.41–3.31 mg.L^−1^).

#### Magnetosomes purity assessment by inductively coupled plasma mass spectrometry (ICP-MS) measurement

2 to 5 mg of extracted magnetosomes from the fourth culture condition were dissolved for 48 h at room temperature with a mix of 70 µL of HCl 37% (w/v) and 572 µL of HNO_3_ 70% (w/v). Samples were then diluted in deionized water to a final volume of 10 mL. Concentrations of elements classified in the ICH-Q3D guidelines, i.e. As, Cd, Hg, Pb, Co, Ni, V, Ag, Au, Ir, Os, Pd, Pt, Rh, Ru, Se, Tl, Ba, Cr, Cu, Li, Mo, Sb, Sn, Al, B, Ca, Fe, K, Mg, Mn, Na, W and Zn, were measured using an inductively coupled plasma-mass spectrometer (Agilent 7900 Quadrupole ICP-MS).

## Results

Here, we present the conditions of preparation of a pharmaceutical cell bank (PCB) which can be used first to store MSR1 magnetotactic bacteria while preserving the properties of these bacteria and second to launch runs of production of pharmaceutical grade magnetosomes. In particular, we demonstrate that the identity, purity and the essential aspects of stability of this strain are preserved during storage.

### Preserved identity, purity, and stability of MSR1 magnetotactic bacteria prepared in a minimal growth medium to yield a pharmaceutically acceptable cell bank.

**Fig. 1:**
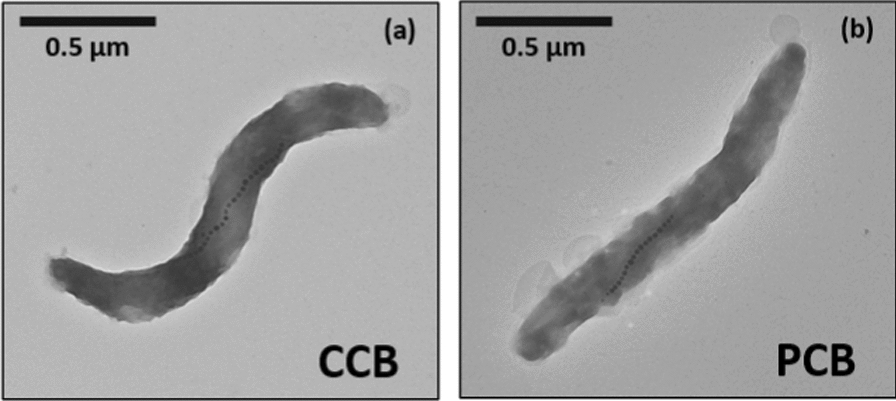
Transmission electron micrographs of a single magnetotactic bacterium originating from the CCB (**a**), or PCB (**b**). Both bacteria display a similar spirilla shape, a similar length of 1.2 µm and the presence of a chain of magnetosomes inside their organism

The CCB (commercial cell bank) was prepared by amplifying MSR1 magnetotactic bacteria in a complete growth medium (“DSMZ—Medium 380,” n.d.) which contains potentially toxic heavy metals and CMR compounds, making the CCB difficult to consider as starting material for bacterial amplifications within a pharmaceutical setting. We therefore prepared a PCB (pharmaceutical cell bank) starting from the CCB, by amplifying MSR1 magnetotactic bacteria in a growth medium devoid of toxic compounds [13, 50]. When we undertook such modifications, which led to the transition from the CCB to PCB, we observed that the identity of the MSR1 bacterial strain was preserved, i.e. bacteria contained in both banks displayed the same characteristics and produced the same desired end-product, i.e. magnetosomes. The preserved identity of magnetotactic bacteria in the PCB is highlighted by the following observations. First, bacteria display a similar average cell length in the two banks, i.e. 1.20 ± 0.25 µm for CCB and 1.21 ± 0.25 µm for PCB, with no significative difference in cell size distribution, i.e. the Mann–Whitney test carried out on such data led to a p value of 0.5823, (Table 1 and Fig. 1). Second, the bacterial morphology appears to be unchanged between both banks, which is revealed by similar cytometry spectra for CCB and PCB bacteria (Additional file 1: Figure S2), and the observation of a spirillum morphology in the TEM images of typical MSR1 bacteria having an optimal magnetic response belonging to both banks (Fig. 1). Third, the number of magnetosomes per cell, N_mag_, and the magnetosome mean size, S_mag_, evolved from N_mag_ = 21.19 ± 5.55 and S_mag_ = 39.99 ± 9.59 nm for CCB, to N_mag_ = 14.44 ± 6.28 and S_mag_ = 37.73 ± 6.71 nm for PCB, indicating that despite a decrease in the number of magnetosomes per cell between CCB and PCB, the size of the magnetosomes remained similar in the two banks (Table 1). This further supports the idea of a preserved strain identity. Fourth, comparison of the bacterial genomes from the two banks to unambiguously confirm the identity of the bacteria produced as part of the characterization criterion of the ICH-Q5e guidelines, which is carried out through PCR amplification of two representative fragments of sizes 209 and 236 pb from MSR1 16S ribosomal sequence, revealed the same bands after gel migration, which is indicative of a similar genomic identity for the two banks (Table 1).

**Table 1:** Table summarizing CCB and PCB characteristics after batch cell bank production in terms of production conditions, identity, and purity: (i) the conditions of preparation for CCB and PCB, *i.e.* medium types and oxygenation, (ii) the parameters used to verify that bacterial identity is maintained while switching from the CCB and PCB, i.e. comparison of cell size, cell morphology, cytometry spectra, number of magnetosomes per cell, mean magnetosome size, magnetosome size distribution, multiplex PCR of MSR1 16S RNA, between CCB and PCB, (iii) the parameters highlighting bacterial purity in PCB and CCB, i.e. bacterial integrity established by cultivating MSR1 bacteria on LB agar plate and observation of a suspension of MSR1 bacteria to verify the absence of contaminants

		CCB	PCB
Production	Medium	Medium 380 (DSMZ)	Minimal medium
	Oxygen concentration	Anaerobic conditions	Microaerobic conditions
Identity	Cell size (µm)	1.20 ± 0.25	1.21 ± 0.25
	Morphology	Spirilla shape with a single magnetosome chain at midcell	Bend shape with a single magnetosome chain at midcell
	Cytometry analysis	Similar size and granulometry	
	Number of magnetosome per cell	21.19 ± 5.55	14.44 ± 6.28
	Mean magnetosome size (nm)	39.99 ± 9.59	37.73 ± 6.71
	Magnetosome size distribution (FWHM)	20.21	11.24
	MSR1 16S RNA Multiplex PCR	2 fragments of 209 and 326 pb Identity confirmed	2 fragments of 209 and 326 pb Identity confirmed
Purity	Integrity on LB agar plate	Free of contaminant	Free of contaminant
	Optic microscopic analysis	Free of contaminant	Free of contaminant

The absence of contaminant in the PCB was demonstrated by growing PCB bacteria in LB agar plates containing a growth medium enabling bacterial growth with a broad-spectrum. No colony was observed after 48 h of incubation at a temperature of 29.5 °C and 37 °C, revealing the purity of the PCB. The results regarding PCB identity and purity assessment compared to CCB are summarized in Table 1.

We then studied the stability of the PCB, which is an essential aspect for being able to use this bank in the long term. First, we studied if the genetic region encoding the proteins responsible for magnetosome production was stable. For that, the genomes of three different preparations of PCB (PCB1, PCB2, PCB3) were compared with the well-known genome of MSR1 (CP027526.1). Although 27, 38 and 82 genomic variations were detected for PCB1, PCB2 and PCB3, they were not considered as significantly affecting the bacterial genomic stability. Indeed, on the one hand no genomic variation was found to occur in PCB with a 100% frequency. On the other hand, genomic mutations occurring with frequencies of 94% and 85% are associated first with single nucleotide polymorphism in the gene ptsI 2 encoding a phosphoenolpyruvate protein phosphotransferase resulting in a missense variation in PC1, and second with a variation in the gene flhF which is involved in flagellar synthesis in PC1. Therefore, the integrity of the genomic region involved in magnetosome mineralization, i.e. the MAI, was fully preserved.

To confirm the long-term preservation of PCB, cell mortality was determined after 4 months of storage at − 80 °C by staining cells with iodide propidium (IP) and determining cell size, granularity and fluorescence using a cytometer technique (Additional file 1: Figure S2). The cell death rate of 21.6% allows a sufficient number of cells to initiate a bacterial growth cycle leading to magnetosome production.

**Fig. 2:**
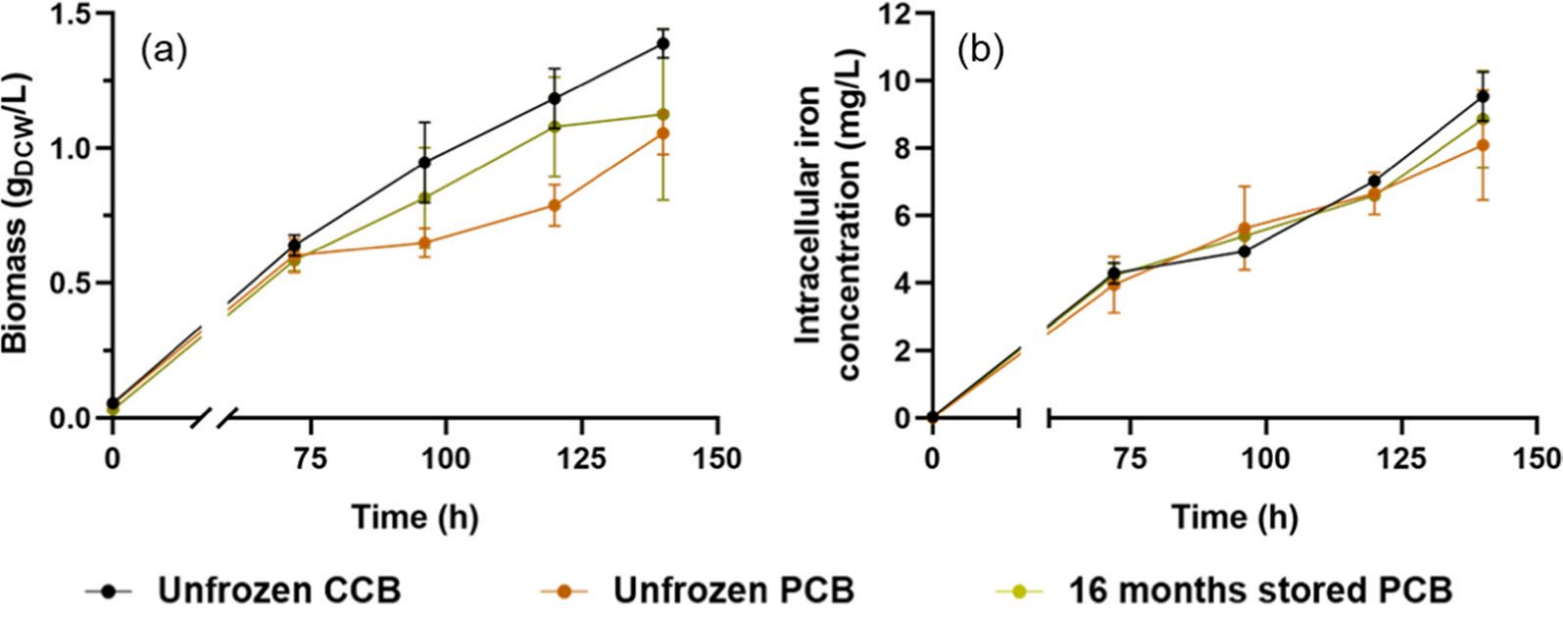
Evolution of the biomass estimated in gram of dry cell weight (DCW) per liter of growth medium (**a**), and intracellular iron concentration (**b**), during the growth step under pH–stat fed-batch culture lasting 140 h, starting the fermentation using unfrozen MSR1 originating from a freshly obtained CCB (unfrozen CCB), unfrozen MSR1 originating from a freshly prepared PCB (unfrozen PCB), unfrozen MSR1 from the PCB stored during 16 months at − 80 °C (16 months stored PCB). The error bar originates from the cultures that were carried out in duplicate

Here, we also show that the PCB creates the conditions for the acclimation of MSR1 magnetotactic bacteria to a minimal growth medium, further enabling bacterial amplification in such medium. For that, we have cultivated unfrozen MSR1 magnetotactic bacteria issued from the PCB and CCB for 140 h (Additional file 1: Figure S4), corresponding to 5.14 ± 0.10 and 5.37 ± 0.11 bacterial generations, respectively. At first sight, the growth of the two types of bacteria, which was carried out in 7.5 L bioreactor using a pH–stat fed-batch method, displayed similar trends, i.e. (i) similar bacterial optical density measured at 565 nm (OD_565_), biomass (DCW), generation time (GT), total quantity of iron internalized in bacteria (TQIIB), volume of feed injected in the culture (VFIC), following 140 h of growth, for unfrozen PCB (OD_565_ = 2.83 ± 0.22, DCW = 5.28 ± 0.39 g, GT = 17.96 ± 1.55 h, TQIIB = 8.10 ± 1.64 mg/L and VFIC = 212 ± 26 mL) and unfrozen CCB (OD_565_ = 3.90 ± 0.28, DCW = 6.76 ± 0.25 g, GT = 18.26 ± 0.26 h, TQIIB = 9.53 ± 0.73 mg/L and VFIC = 235 ± 6 mL) (Fig. 2 and Table 2) and (ii) similar increases of the biomass and quantity of iron internalized in bacteria during bacterial growth (Fig. 2). However, interestingly, magnetosome production at 140 h was significantly larger for the PCB than for the CCB, i.e. the magnetosome mass (MM), the magnetosome volumetric yield (MVY), total intracellular iron as magnetosomes (TIIM), and number of magnetosomes per cells (NMC), display higher values for PCB (MM = 24.90 ± 5.66 mg, MVY = 4.98 ± 1.13 mg/L, TIIM = 44.38 ± 1.14%, NMC = 24.15 ± 13.18) than for CCB (MM = 7.05 ± 0.64 mg, MVY = 1.45 ± 0.13 mg/L, TIIM = 9.26 ± 0.49%, NMC = 12.11 ± 4.79), without leading to a significant change in magnetosome sizes, i.e. SM = 36.94 ± 6.07 for PCB and SM = 35.16 ± 6.94 for CCB. The magnetosome size, which is essential to yield magnetosome ferrimagnetic properties and chain arrangement, is preserved in the PCB. Furthermore, the acclimatization of the MSR1 strain to the minimal growth medium that the PCB enables to achieve, leads to a higher conversion rate of the internalized iron into magnetosomes and to 3.4 times larger magnetosome production in the PCB than in the CCB (Table 2). In other words, without being acclimated to the minimal growth medium, the MSR-1 strain leads to a less efficient transformation rate of internalized iron into magnetosomes.

**Table 2:** Summary of bacterial growth parameters and magnetosome properties obtained after 140 h of cultivation, corresponding to ~ 5 bacterial generation, in a 7.5 L bioreactor fed-batch culture of MSR1 bacteria derived from unfrozen CCB, unfrozen PCB and 16-month stored PCB to evaluate PCB stability after long-term storage and compare production performance with CCB, i.e. optical density of bacterial suspension measured at 565 nm (OD_565_), biomass (g), generation time (h), total intracellular iron in mg of intracellular iron per liter of growth medium, quantity of internalized iron inside cells in mg per gram of dried cell weight, volume of feed medium injected during culture, mass of dried magnetosomes purified, yield of magnetosome production in mg of dried magnetosomes purified per liter of growth medium, total intracellular iron in the form of magnetosome in percentage and number of magnetosomes per bacterium, magnetosome size (nm)

Starting culture inoculum	Unfrozen CCB	Unfrozen PCB	16 months stored PCB
OD_565_	3.90 ± 0.28	2.83 ± 0.22	2.91 ± 1.16
Biomass (g)	6.76 ± 0.25	5.28 ± 0.39	5.64 ± 1.59
Generation time (h)	18.26 ± 0.26	17.96 ± 1.55	15.71 ± 0.31
Total intracellular iron concentration (mg/L)	9.53 ± 0.73	8.10 ± 1.64	8.86 ± 1.44
Iron ratio per gram of DCW (mgFe/g)	6.87 ± 0.27	7.64 ± 0.98	8.39 ± 3.66
Volume of feed injected (mL)	235 ± 6	212 ± 26	235 ± 5
Magnetosome mass (mg)	7.05 ± 0.64	24.90 ± 5.66	28.40 ± 14.14
Magnetosome volumetric yield (mg/L)	1.45 ± 0.13	4.98 ± 1.13	5.66 ± 2.82
Total intracellular iron as magnetosome (%)	9.26 ± 0.49	44.38 ± 1.14	38.65 ± 14.88
Number of magnetosome per cell	12.11 ± 4.79	24.15 ± 13.18	21.01 ± 9.43
Magnetosome size (nm)	35.16 ± 6.94	36.94 ± 6.07	35.50 ± 7.11

The effect of bacterial storage after 16 months at − 80 °C on the stability of the MSR-1 strain is also examined by comparing the parameters of bacterial growth and magnetosome production between the unfrozen and frozen MSR1 strains originating from PCB. These parameters appear to be very similar for both conditions (Table 2 and Fig. 2), indicating that the capacity of MSR-1 bacteria to grow and produce magnetosomes under our well-established conditions involving pre-growth and growth steps are preserved during storage.

Finally, we determined whether the stability of the MSR1 strain in PCB is maintained over many bacterial generations of 100, which is largely sufficient to allow magnetosome production using our three-step bacterial amplification method involving typically 16 to 17 generations (Additional file 1: Figure S4). After having cultivated bacteria belonging to the PCB over 100 generations in the presence of frequent exposure to high oxygen concentration, we observed that the essential parameters characterizing the bacteria and magnetosomes remained unchanged, i.e. the bacterial cell length is 1–1.5 µm, a maximum magnetic response is observed in all bacteria, the number of magnetosomes per cell remains between 8.90 ± 4.02 and 19.78 ± 6.60 (Table 3 and Additional file 1: Figure S5), that is to say there are enough magnetosomes to ensure an optimal magnetic response of the cells and the magnetosome mean size that has slightly decreased over 100 generations remains sufficiently large (> 20 nm) to preserve the most essential magnetosome magnetic property (Table 3). Therefore, despite frequent exposures of MSR1 bacteria to high oxygen concentrations during growth, magnetotactic bacteria originating from PCB maintain a persistent capacity of being able to produce magnetosomes over 100 generations of MTB amplifications. It allows us to consider the use of PCB for bacterial amplification and associated magnetosome mass-production using large fermentation volumes requiring an important number of bacterial generations.

**Table 3:** Parameters enabling the assessment of bacterial stability and magnetosome production over 100 generations for PCB-derived MSR1 bacteria after 3–5 days of subculture in flask for 101 days

	Cell size (µm)	Morphology	Number of magnetosome per cell	Mean magnetosome size (nm)
1st Generation	1.21 ± 0.25	Bend shape Single magnetosome chain at midcell	14.44 ± 6.28	37.73 ± 6.71
20th Generation	1.24 ± 0.16	Bend shape Single magnetosome chain at midcell	8.90 ± 4.02	33.50 ± 7.09
40th Generation	1.23 ± 0.27	Bend shape Single magnetosome chain at midcell	9.15 ± 3.83	28.83 ± 7.74
100th Generation	1.27 ± 0.28	Bend shape Single magnetosome chain at midcell	19.78 ± 6.60	24.58 ± 5.24

Such parameters are cell size (µm), cell morphology, number of magnetosomes per cell and mean magnetosome size (nm)

### Highly pure magnetosomes originating from PCB

**Fig. 3:**
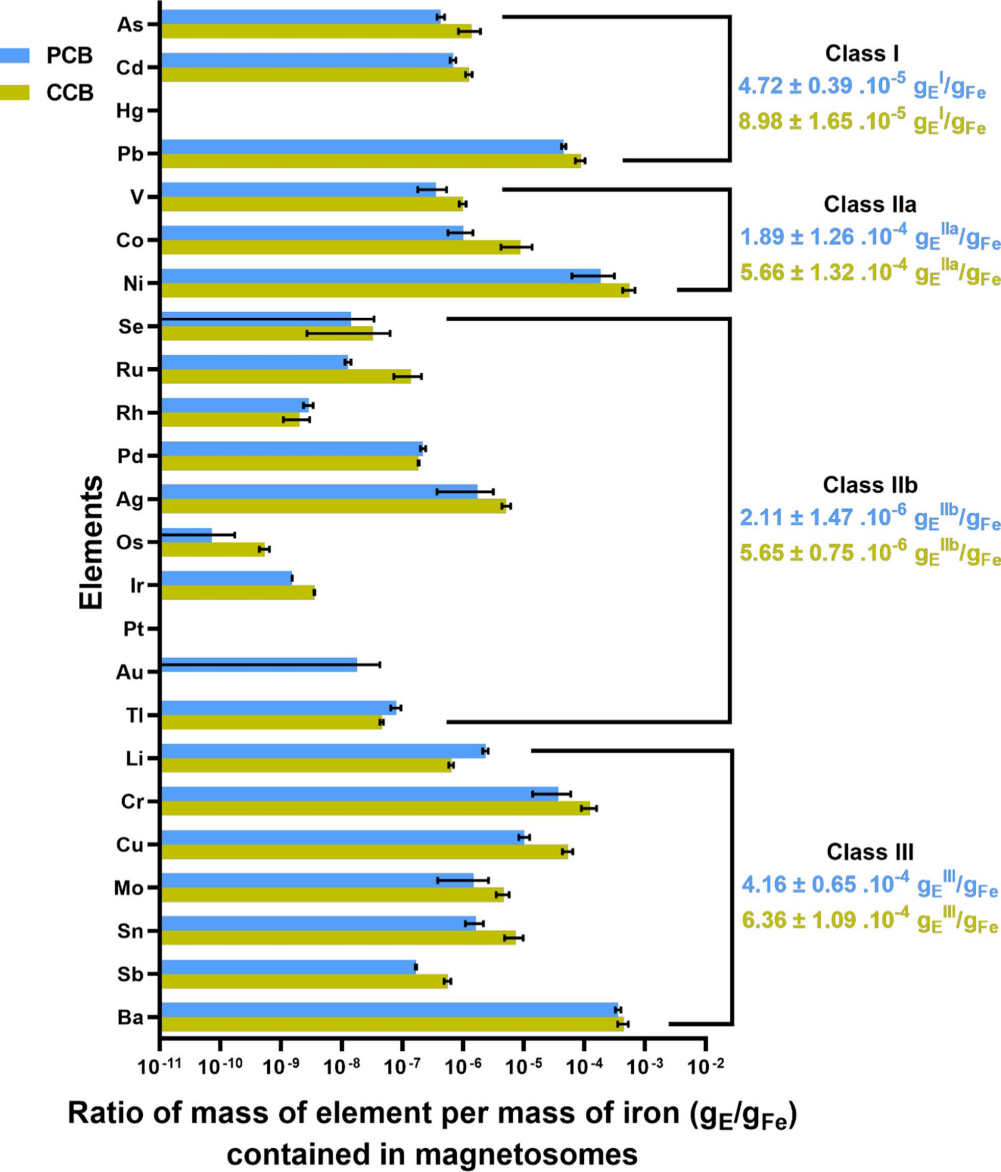
Histograms indicating the mass of each metallic element from toxic classes I, IIa, IIb and III containing for 1 g of iron contained in magnetosomes originating from CCB and PCB. Elements are regrouped in classes according to ICH-Q3D standards and green and blue values indicates the cumulative amount of each element in a given class of the mass of each metallic element containing for 1 g of iron

We then studied the purity level of magnetosomes produced with CCB and PCB in a minimal growth medium. As shown in Fig. 3, magnetosomes issued from PCB possess a higher iron purity level considering toxic elements belonging to classes I to III, defined as ΣM_i_/M_Fe_, where M_i_ and M_Fe_ are the masses of all heavy metals comprised in magnetosomes and of iron, respectively, which is ΣM_i_/M_Fe_ = 99.935%_Fe_, 99.870%_Fe_ of iron using CCB and PCB as inoculum. The iron purity level decreases to ∼ 93.6%_Fe_ using the medium of Zhang et al. [76] which include more metallic compounds and non-pharmaceutical grade products. This clearly indicates a large improvement in magnetosome purity achieved with the PCB compared with magnetosomes produced using CCB or in non-depleted growth media [13]. Furthermore, considering the less biocompatible metallic compounds in class I according to ICH-Q3D standard, their concentration in magnetosomes is very low, i.e. the average concentration of As, Cd, Hg and Pb was 4.72 ± 0.39 10^–5^ g/g_Fe_ with a 47.4% reduction compared to CCB-derived magnetosome content (Fig. 3). Considering parenteral administration of magnetosomes for cancer treatment, the recommended cumulative maximal concentration of class I metals is 25 µg/day [18]. When we apply this rule to magnetosomes, it results in a maximum daily injectable therapeutic dose of 530 mg in iron of magnetosomes. For a typical dose of 50 mg in iron of magnetosomes^1^, that we plan to administer in a localized prostate tumor of Gleason 7 of typical diameter 1 to 2 cm, the average quantity class I metals contained in magnetosomes in average is 2.36 ± 0.20 µg, which is more than 10 times below the maximum cumulative daily injectable dose of these metals of 25 µg.

## Discussion

Here, we have determined conditions for producing a MSR1 pharmaceutical cell bank, i.e. a cell bank containing magnetotactic bacteria amplified under pharmaceutically acceptable conditions with preserved identity, purity and stability compared with the commercial cell bank (CCB), leading to the production of highly pure magnetosomes, i.e. essentially devoid of other heavy toxic metals than iron listed in the ICH-Q3D standards.

To achieve this aim, we used a series of optimal growth conditions during the growth. First, during the growth step, we bubbled oxygen in the growth media at 20 mbar, i.e. ∼ 2% vol., to favor MSR1 growth up while avoiding exceeding these values above which magnetosome production would be prevented [28, 56]. Second, we fixed the iron concentration at 100 µM to maximize biomass and magnetosome production [59]. Third, we recovered cells in an optimal physiologic state at the end of the growth phase at 142 h, i.e. after the MTB growth phase would stop amplifying. Fourth, we removed from the standard MSR1 magnetotactic bacterium growth medium the compounds that are considered as potentially toxic according to ICH-Q3D standards (As, Cd, Hg, Pb, Co, Ni, V, Ag, Au, Ir, Os, Pd, Pt, Rh, Ru, Se, Tl, Ba, Cr, Cu, Li, Mo, Sb, and Sn) to end up with a minimal growth medium essentially containing Cl, Mg, K, Fe and Ca that we successfully used to amplify MSR1 magnetotactic bacteria.

Interestingly, the method that we employed enabled to avoid mutations in the MAI locus responsible for magnetosome formation in MSR1 [70], hence enabling to overcome the difficulties associated with the frequent mutations of MSR1 associated with oxidative stress [54, 71]. Our well-controlled conditions of amplification and low medium oxygenation may favor the decrease of reactive oxygen species and participate in the reduction of oxidative stress via production of magnetosomes which are known to be able to decrease reactive oxygen species [26].

Once bacterial amplification conditions were established, as described above, it was ensured that the MSR1 strain prepared under these conditions could be cryo-preserved at a bacterial concentration that is sufficiently large to allow the launch of batches of magnetosome production [23, 64] as part of a bioprocess intensification method to reduce duration and cost of seed-train [73]. For that, we have established successful high cell load cryo-preservation conditions of the MSR1 strain with an OD_565_ of 1 of the inoculums and to 9.10^8^ MTB per mL, which corresponds to a 10 times larger bacterial concentration compared with the concentration of the commonly starting inoculum used in commercial cell banks (DSMZ 6361).

Finally, while the properties of MTBs and their magnetosomes can change depending on the used culture conditions, in particular by varying oxygenation or composition of the culture media [13, 50, 56], we determined that the PCB preserves the purity and identity of MSR1, with the production of cuboctahedral magnetosomes organized as a single chain and having a large size (> 20 nm) sufficient to concede an optimal magnetic response. In addition, the stability of the strain issued from the PCB was studied by amplifying MTB over 100 generations, inducing several cycles of cytokinesis that are believed to be responsible for magnetosome dissemination [30, 66] not resulting in any major changes of the magnetosome properties mentioned above. Other studies have shown that MTB bacterial amplification undertaken under different conditions than ours could yield genetic modifications of the MSR1 strain characterized by the deletion of mms and mam genes involved in crystal maturation in the MAI [54, 70, 71]. Interestingly, our conditions lead to much less drastic genetic changes, the only genetic variations that we observed occurring at a low occurrence rate of 94 and 85%, first as a single nucleotide polymorphism in the gene ptsI 2 encoding a phosphoenolpyruvate protein phosphotransferase [69], and second as an insertion in the gene flhF which encodes the key protein FlhF required for complete flagellar synthesis [38, 75]. Furthermore, none of these modifications affected the genes responsible for magnetosome production in the MAI genetic region, therefore resulting in no apparent impact on magnetosome fabrication.

We attribute the successful 3.4-fold magnetosome yield improvement during fed-batch production cultivation to the adaptation of MTB issued from PCB to the minimal growth medium. MTB originating from the PCB were first amplified in the minimal growth medium during production. The strain adapted itself to the minimal growth medium presumably by following the adaptative laboratory evolution (ALE) [44], in a similar manner as observed for other strains such as *Geobacter sulfurreducens* or *Escherichia coli* that resulted, after serial cultivations in a minimal medium, in an increased production of biomass and bacterial products of interest [57, 67]. In addition, storage at − 80 °C over 16 months did not affect the increased production performances with PCB and successfully remained stable according to the high cell-load cryopreservation strategy that was developed.

In our study, we haven’t only reduced the number of compounds in the growth medium, but we have also shown a direct impact of the composition of such a medium in the magnetosome composition. In other words, the PCB that we have developed has the double advantage of being prepared in a minimal, non-toxic medium and of producing highly pure magnetosomes in iron. We drew our inspiration from a well-known property of MTB concerning the incorporation of other metals than iron such as Mn, Zn, Cu and Co inside magnetosomes, which is achieved by enriching MTB growth medium in these compounds [49, 68]. Here we have carried out the experiment in reverse direction, i.e. we have reduced the composition of the MTB growth medium in other metals other than iron, to eliminate as much as possible their presence inside magnetosomes. The use of the PCB as starting inoculum allows the generation of highly pure magnetosomes in iron relatively to other metals, i.e. magnetosomes with a purity in iron of 99.935%_Fe_. The level of purity is much higher than that obtained with the standard medium from Zhang et al. [76] leading to ∼ 93.6%_Fe_ of iron per magnetosome or with a depleted minimal medium starting from the CCB resulting in ∼ 99.8%_Fe_ of iron per magnetosome [13].

While in terms of metallic composition, magnetosomes are characterized by the presence of an iron oxide core, in the absence of a specific treatment, some other non-metallic and non-toxic elements have been observed in small quantities (< 1.5 10^–4^%_Fe_ per element in average) such as Na, Ca, Mg and K which could under their cationic form surround the negatively charged magnetosome surface [46]. Such impurities in the magnetosome membrane could be eliminated by removing the magnetosome membrane from the inorganic core to improve the particle purity [40, 50].

## Conclusion

Our study demonstrates the successful development of a *Magnetospirillum gryphiswaldense* MSR1 pharmaceutical cell bank, characterized by the same identity as the original MTB, a purity highlighted by an absence of contaminants, and a stability over 100 generations of MTB amplification, or following cryo-preservation after 16 months of storage. In addition, the realization of the PBC in a minimal growth medium enables to acclimate MTB to such medium, which in turn results in the production of highly pure magnetosomes containing an average of 99.935% of iron per magnetosome. Furthermore, the PCB is achieved under high cell load conditions of 9.10^8^ cells/mL, which do not appear to be detrimental to bacterial survival and enables the direct launch of a large-scale magnetosome production. Overall, our study presents the conditions for developing a dedicated cell bank of MSR1 strain adapted for the production of pharmaceutically compatible magnetosome dedicated to biomedical applications in the context of magnetosome industrial production and commercial sales.

### Supplementary Information

**Additional file 1: Figure S1.** Variation of the partial pressure of oxygen (pO_2_) and pH of the pharmaceutical cell bank minimal medium during the 142 hours of the growth step of MSR1 magnetotactic bacteria originating from the CCB. Red arrows indicate times at which the culture was sparged with a gas mix of O_2_/N_2_ (2/98 %). Stirring and temperature were maintained at 110 rpm and 29.5 °C. Initial (at 0 hour) and final (at 140 h) bacterial optical density measured at 565 nm (OD 565 nm) and bacterial magnetic response are specified in the inset table. **Figure S2.** **a** Cytograms of living and inactivated MSR1 magnetotactic bacteria originating from the CCB and PCB, displaying the FSC (forward scatter) signal as a function of the SSC (side scatter) signal, where the FSC and SSC signals represent the distributions of bacterial size and granulometry, respectively. The coloration represents the density of MSR1 counts according to FSC and SSC with red for the highest and blue the lowest density. **b** Cytogram of unfrozen CCB cells and 4 months PCB cells stored at – 80 °C, which were made fluorescent using IP labelling. Cells are considered as dead above a signal of 10^3^ arbitrary unit. **Figure S3**. Variation of the partial pressure of oxygen (pO_2_), airflow in mL of air bubbled in the growth medium per minute, stirring of the growth medium in rotation per minute, and pH of the growth medium during the 140 h of the growth step of MSR1 magnetotactic bacteria originating from the PCB. During the entire cultivation, pH was kept at 6.84 (yellow) and oxygen partial pressure (red) was monitored to maintain microaerobic conditions were maintained. Stirring and temperature were maintained at 110 rpm and 29.5 °C, respectively. **Figure S4.** Histogram showing the number of cumulative bacterial generations achieved at the end of the first pre-growth step (PC1) in 250 mL squared flask bottles, at the end of the second pre-growth step (PC2) in a 3 L bioreactor and at the end of the growth step during pH-stat fed-batch culture in a 7.5 L bioreactor (FBC).

## Data Availability

Data presented in this article are available on demand.

## Footnotes

1 The human dose of 50 mg was estimated as an extrapolation of the magnetosome dose leading to full tumor disappearance in mice bearing PC3-Luc prostate tumors of 150 mm^3^ administered with 3 µg/mm^3^ of magnetosomes, having their tumors heated 10 times at 45 °C during 10 minutes (manuscript in preparation).
