# Supplementary material for: Set-up of a pharmaceutical cell bank of Magnetospirillum gryphiswaldense MSR1 magnetotactic bacteria producing highly pure magnetosomes
Source: Microb Cell Fact. 2024 Feb 28;23:70. doi: 10.1186/s12934-024-02313-4 (PMC10903015; doi:10.1186/s12934-024-02313-4)
Supplement: Supplementary file 1 — Additional file 1: Figure S1. Variation of the partial pressure of oxygen (pO2) and pH of the pharmaceutical cell bank minimal medium during the 142 hours of the growth step of MSR1 magnetotactic bacteria originating from the CCB. Red arrows indicate times at which the culture was sparged with a gas mix of O2/N2 (2/98 %). Stirring and temperature were maintained at 110 rpm and 29.5 °C. Initial (at 0 hour) and final (at 140 h) bacterial optical density measured at 565 nm (OD 565 nm) and bacterial magnetic response are specified in the inset table. Figure S2. a Cytograms of living and inactivated MSR1 magnetotactic bacteria originating from the CCB and PCB, displaying the FSC (forward scatter) signal as a function of the SSC (side scatter) signal, where the FSC and SSC signals represent the distributions of bacterial size and granulometry, respectively. The coloration represents the density of MSR1 counts according to FSC and SSC with red for the highest and blue the lowest density. b Cytogram of unfrozen CCB cells and 4 months PCB cells stored at – 80 °C, which were made fluorescent using IP labelling. Cells are considered as dead above a signal of 103 arbitrary unit. Figure S3. Variation of the partial pressure of oxygen (pO2), airflow in mL of air bubbled in the growth medium per minute, stirring of the growth medium in rotation per minute, and pH of the growth medium during the 140 h of the growth step of MSR1 magnetotactic bacteria originating from the PCB. During the entire cultivation, pH was kept at 6.84 (yellow) and oxygen partial pressure (red) was monitored to maintain microaerobic conditions were maintained. Stirring and temperature were maintained at 110 rpm and 29.5 °C, respectively. Figure S4. Histogram showing the number of cumulative bacterial generations achieved at the end of the first pre-growth step (PC1) in 250 mL squared flask bottles, at the end of the second pre-growth step (PC2) in a 3 L bioreactor and at the end of the growth step during pH- [file 12934_2024_2313_MOESM1_ESM.docx]

**Set-up of a stable *Magnetospirillum gryphiswaldense* MSR1 cellular bank to produce pharmaceutical grade magnetosomes**

Théo Chades^1,2^, Raphaël Le Fèvre^1^, Imène Chebbi^1^, Karine Blondeau^2^, François Guyot^3^, Edouard Alphandéry^1,3,4^

^1^ Nanobacterie SARL, 36 Boulevard Flandrin, 75116, Paris. The affiliation of Nanobacterie includes the two subsidiaries of Nanobacterie: AlphaOnco Lux, 16 Avenue Pasteur L-2310 Luxembourg and AlphaOnco Swiss, Route de l'Ile-au-Bois 1a, 1870, Monthey, Switzerland.

^2^ Institut de biologie intégrative de la cellule, UMR 9198, Université Paris Saclay, 1 Av. de la Terrasse, 91198, Gif sur Yvette, France.

^3^ Institut de minéralogie de physique des matériaux et de cosmochimie UMR 7590, Sorbonne Université, Université Pierre et Marie Curie, Muséum National d’Histoire Naturelle, 4 Place Jussieu, 75005, Paris, France.

^4^ Institute of Anatomy, UZH University of Zurich, Institute of Anatomy, Winterthurerstrasse 190, CH-8057, Zurich, Switzerland.

* Corresponding author e-mail: [edouardalphandery@hotmail.com](mailto:edouardalphandery@hotmail.com)

**Additional Figures**

Additional file 1: Figure S1. Variation of the partial pressure of oxygen (pO_2_) and pH of the pharmaceutical cell bank minimal medium during the 142 hours of the growth step of MSR1 magnetotactic bacteria originating from the CCB. Red arrows indicate times at which the culture was sparged with a gas mix of O_2_/N_2_ (2/98 %). Stirring and temperature were maintained at 110 rpm and 29,5°C. Initial (at 0 hour) and final (at 140 hours) bacterial optical density measured at 565 nm (OD 565 nm) and bacterial magnetic response are specified in the inset table.

Additional file 1: Figure S2. (a), Cytograms of living and inactivated MSR1 magnetotactic bacteria originating from the CCB and PCB, displaying the FSC (forward scatter) signal as a function of the SSC (side scatter) signal, where the FSC and SSC signals represent the distributions of bacterial size and granulometry, respectively. The coloration represents the density of MSR1 counts according to FSC and SSC with red for the highest and blue the lowest density. (b), Cytogram of unfrozen CCB cells and 4 months PCB cells stored at -80°C, which were made fluorescent using IP labelling. Cells are considered as dead above a signal of 10^3^ arbitrary unit.

Additional file 1: Figure S3. Variation of the partial pressure of oxygen (pO_2_), airflow in mL of air bubbled in the growth medium per minute, stirring of the growth medium in rotation per minute, and pH of the growth medium during the 140 hours of the growth step of MSR1 magnetotactic bacteria originating from the PCB. During the entire cultivation, pH was kept at 6.84 (yellow) and oxygen partial pressure (red) was monitored to maintain microaerobic conditions were maintained. Stirring and temperature were maintained at 110 rpm and 29,5 °C, respectively.

Additional file 1: Figure S4. Histogram showing the number of cumulative bacterial generations achieved at the end of the first pre-growth step (PC1) in 250 mL squared flask bottles, at the end of the second pre-growth step (PC2) in a 3L bioreactor and at the end of the growth step during pH-stat fed-batch culture in a 7.5L bioreactor (FBC).


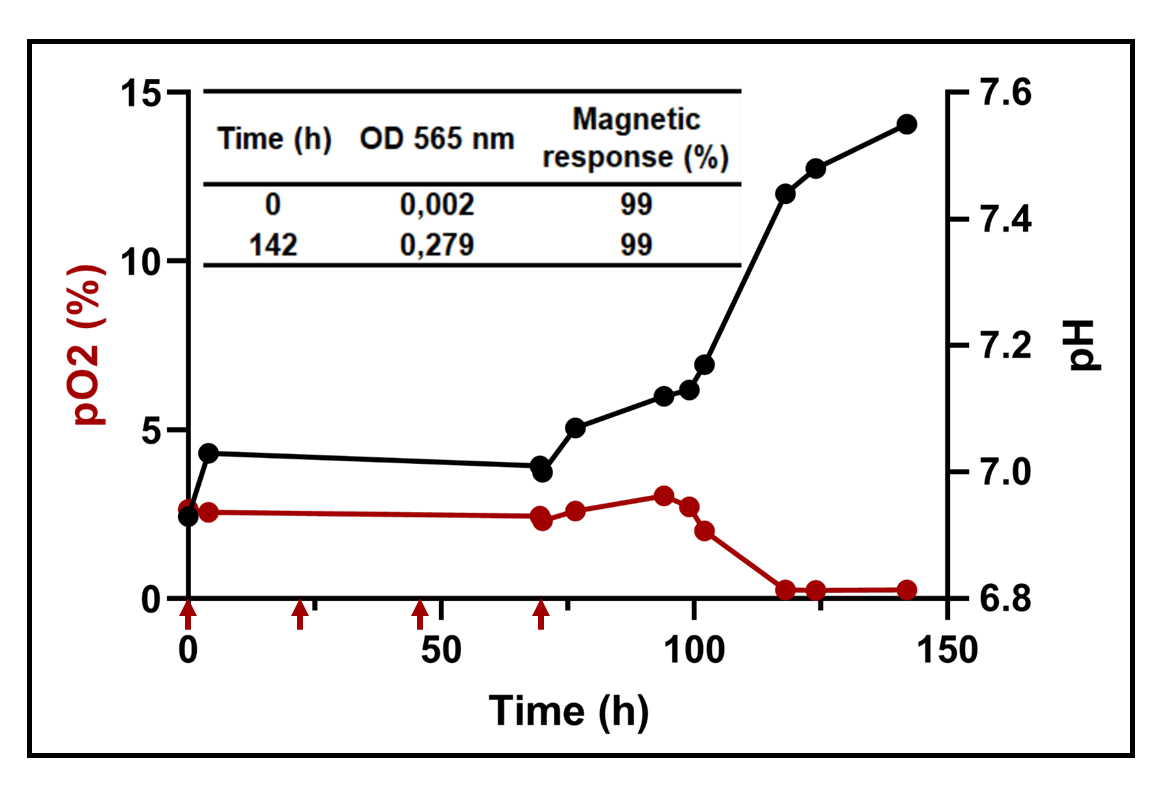


Suppl. Fig. 1


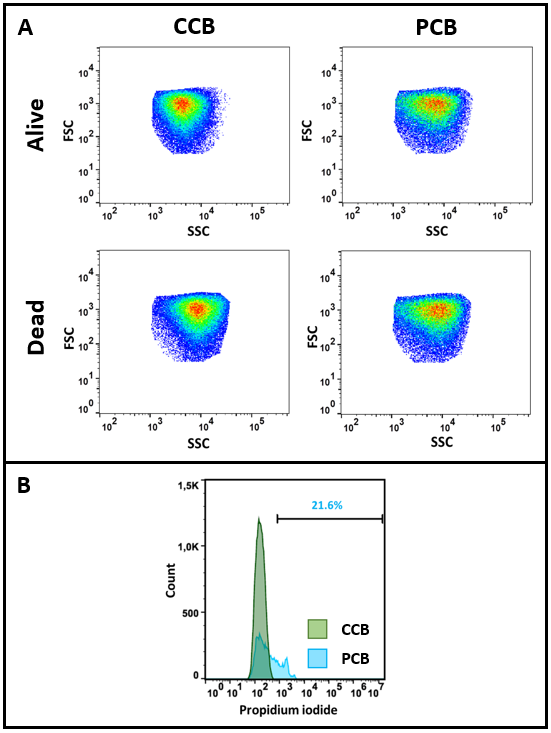


Suppl. Fig. 2


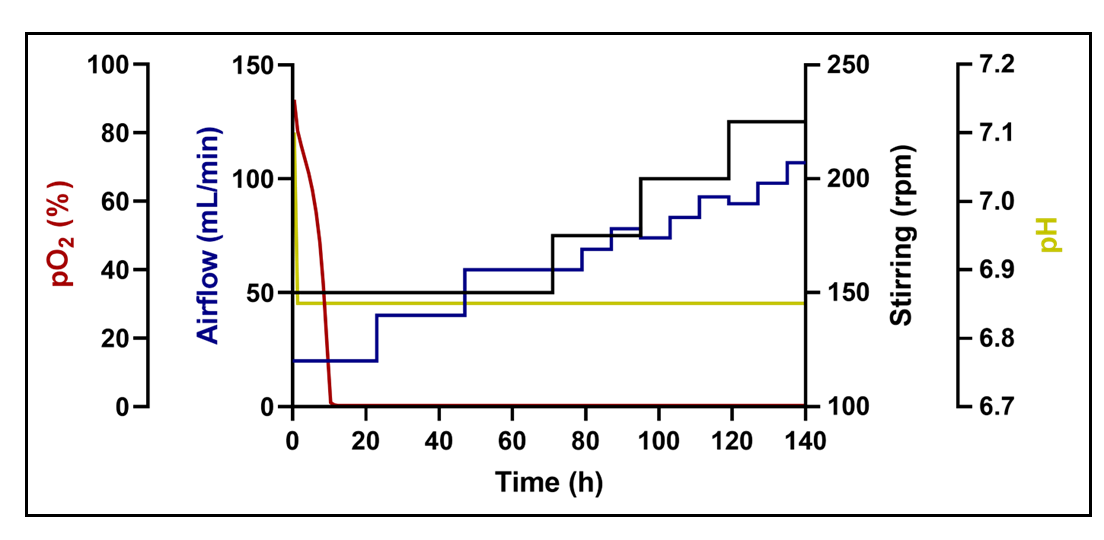


Suppl. Fig. 3


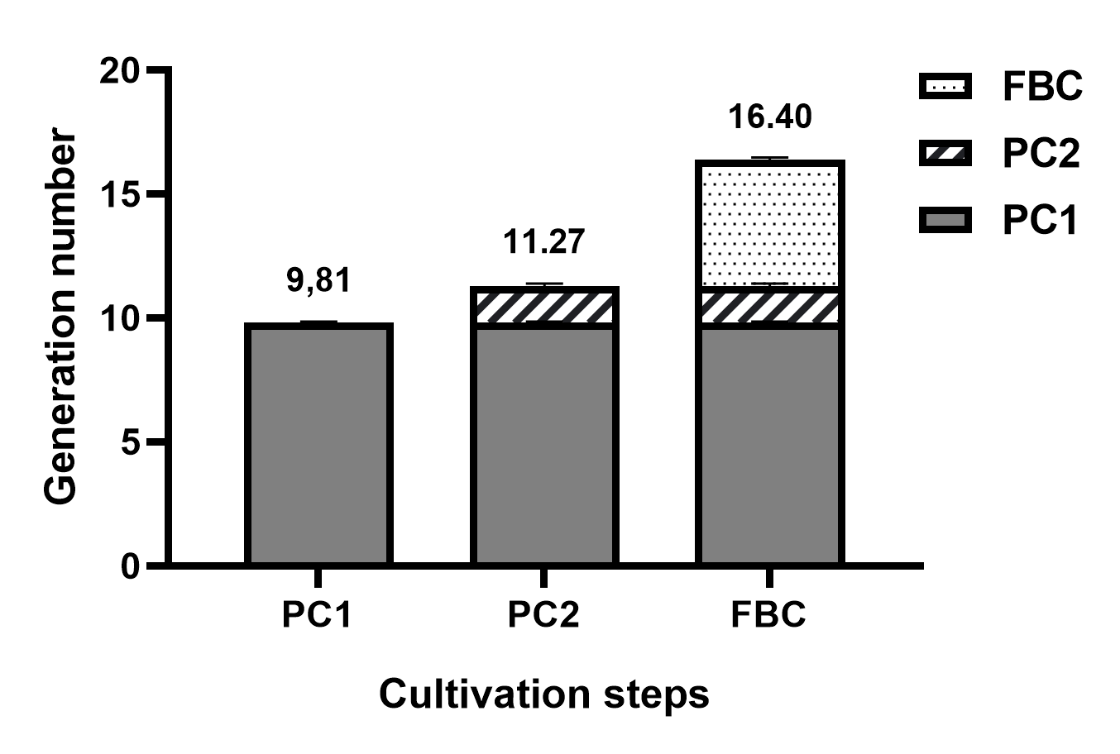


Suppl. Fig. 4
